# Cerebral Abscess Mimicking Intracerebral Hemorrhage: A Case Report

**DOI:** 10.7759/cureus.82744

**Published:** 2025-04-21

**Authors:** Patricia Anne I Batol, Steven G Villaraza

**Affiliations:** 1 Department of Neurology, Jose R. Reyes Memorial Medical Center, Manila, PHL

**Keywords:** brain abscess, cerebral abscess, hemorrhagic brain abscess, intracerebral hemorrhage, pyogenic brain abscess

## Abstract

Hemorrhagic pyogenic cerebral abscesses are a rare clinical entity that can present diagnostic challenges and lead to treatment delays. We report a case of a 46-year-old female who initially presented with a sudden onset of focal neurological deficits, raising a suspicion of a cerebrovascular etiology. Further diagnostic workup revealed a hemorrhagic pyogenic brain abscess. The patient was managed successfully with a conservative approach, receiving medical treatment after declining surgical intervention, and was discharged with only minimal neurological residuals. Although hemorrhagic pyogenic brain abscesses are uncommon, maintaining a high index of suspicion, supported by comprehensive history taking and targeted diagnostic testing, is imperative for early detection and effective management to mitigate potential morbidity and mortality.

## Introduction

A cerebral abscess is a focal area of infection within the brain parenchyma, characterized by necrosis and inflammation or cerebritis resulting from an infectious process by hematogenous, direct spread, or iatrogenic causes [1]. Hemorrhage among cerebral abscesses is rare and may cause diagnostic dilemmas [2]. Here, we report a case of a hemorrhagic pyogenic cerebral abscess that presented clinically and radiographically as an intracerebral hemorrhage.

## Case presentation

A 46-year-old woman with no known comorbidities presented with a sudden onset of right-sided weakness, headache, and loss of verbal output. Vital signs upon admission were within normal limits. On neurologic examination, the patient was awake, with right homonymous hemianopsia, Broca’s aphasia, and right hemiparesis with a motor strength grade of 2.

**Figure 1:**
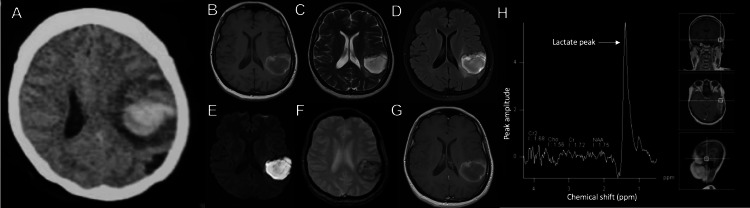
Imaging findings A: Non-contrast cranial computed tomography (CT) scan showing an irregularly shaped hyperdense lesion with surrounding vasogenic edema, measuring 6.1 × 3.8 × 5.1 cm (cranio-caudal × width × anteroposterior), in the left temporoparietal region. B: T1-weighted magnetic resonance imaging (MRI) showing a multiloculated, irregularly shaped hyperintense rim with an iso- to hypointense center in the left temporoparietal area. C & D: T2-weighted and fluid-attenuated inversion recovery (FLAIR) MRI sequences showing prominent surrounding vasogenic edema. E: Diffusion-weighted MRI sequence showing hyperintense signals due to restricted diffusion at the said area. F: Gradient echo images showing dark blooming signals. G: Post-gadolinium axial T1-weighted image showing ring enhancement. H: MR spectroscopy demonstrating decreased N-acetylaspartate (NAA) and choline with a 1.3 parts per million lactate peak.

Initial electrocardiogram, blood count, biochemical tests, and coagulation parameters were within normal limits. A non-contrast cranial computed tomography (CT) scan revealed a hyperdense focus at the left temporoparietal area consistent with a lobar hemorrhage (Figure 1A). However, what was notable was the presence of marked vasogenic edema surrounding the hyperdensity, leading the clinician to consider an underlying structural pathology; hence, gadolinium-enhanced cranial magnetic resonance imaging (MRI) was requested. The contrast-enhanced MRI revealed a heterogeneous hyperintense focus on T1-weighted imaging (Figure 1B), hyperintense on T2-weighted imaging (Figure 1C), with surrounding vasogenic edema on fluid-attenuated inversion recovery (FLAIR) imaging (Figure 1D), hyperintense on diffusion-weighted imaging (Figure 1E), dark blooming signals on gradient echo (Figure 1F) at the left temporoparietal area and ring enhancement on post gadolinium studies (Figure 1G). MR angiography and venography were likewise requested but were unremarkable. Magnetic resonance spectroscopy (MRS) revealed decreased N-acetyl aspartate (NAA) and choline with a 1.3 parts per million lactate peak (Figure 1H). The radiologic findings suggested a pyogenic cerebral abscess; therefore, empiric antibiotic therapy with vancomycin, metronidazole, ceftriaxone, and corticosteroids was initiated.

Following a thorough evaluation and exclusion of other potential sources, chronic suppurative otitis media was determined to be the most probable focus of infection. Specimen from the ear discharge was collected for culture and sensitivity studies, which revealed growth of *Streptococcus spp*. Blood culture yielded negative results. 2D echocardiography with Doppler studies revealed no valvular pathologies. Neurosurgical intervention was offered for definitive management. However, the patient refused. Parenteral culture-guided antibiotic treatment was continued for eight weeks. Surveillance neuroimaging after two weeks revealed interval resolution of the abscess. The patient was eventually discharged with minimal right-sided residual deficits, a Modified Rankin Scale (mRS) score of 2, and a motor strength grade of 4.

## Discussion

A cerebral abscess is a focal infectious collection of necrotic tissue within the central nervous system [1,2]. It comprises approximately 8% of intracranial masses in developing countries and 1% in Western countries [1-3]. Cerebral abscesses are usually polymicrobial, wherein the most common pathogen would include gram-positive bacteria such as *Streptococcus spp.* (30-60% of cases), and *Staphylococcus spp.* (10 to 20% of cases), and enteric gram-negative bacteria (23 to 33% of cases) [1,3,4].

Cerebral abscesses typically arise from the contiguous spread of pathogens in immunocompetent individuals. This occurs most frequently from local foci such as otitis media, mastoiditis, sinusitis, neurosurgical procedures, and head trauma, collectively accounting for approximately 40% to 50% of all reported cases. The pathophysiological process involves the direct extension of infection from these local foci into the central nervous system, often facilitated by the proximity of anatomical structures or post-surgical alterations to the blood-brain barrier [1,2,4].

Beyond contiguous spread, cerebral abscesses may also emerge from distant infectious foci, including dental infections or pulmonary abscesses. While less common, these conditions represent significant risk factors, as pathogens from these sites can embolize through the bloodstream, eventually seeding the brain and leading to abscess formation [1,2,3,5].

In rare cases, hematogenous dissemination serves as the source of cerebral abscesses. This can occur in patients with underlying systemic conditions that predispose them to bacteremia, such as congenital heart disease, pulmonary arteriovenous malformations, or hereditary hemorrhagic telangiectasia. In these cases, the pathogens are carried via the bloodstream, bypassing the pulmonary circulation and directly reaching the brain, where they can initiate infection [2,3,5].

Overall, the development of cerebral abscesses in immunocompetent individuals underscores the complex interplay between local and systemic infectious processes, with both contiguous spread and hematogenous dissemination playing key roles in the pathogenesis of these life-threatening conditions [2,3].

Symptoms of cerebral abscesses may be indolent to fulminant and nonspecific, ranging from headaches, mental status changes, focal neurologic deficits, fever, and seizures [1,2]. Headache is the predominant symptom, accounting for 69% of patients, followed by fever (43%) and other focal neurologic deficits (48%). However, the location of the abscess significantly influences the patient's neurological presentation [1-4].

Imaging findings in non-contrast CT scans can be seen as an irregular hypodense area that may exhibit patchy enhancement. As the cerebritis progresses, a distinct ring enhancement becomes visible. Although non-contrast CT can identify a possible cerebral abscess, MRI with concurrent spectroscopy is the imaging of choice due to its high sensitivity in detecting early cerebritis up to the late capsule stage. It may also estimate the necrosis, lesion extent, and edema surrounding the lesion [1,2,6].

The exact incidence of a hemorrhagic cerebral abscess remains unknown due to the rarity of the case; however, there have been several reported cases. The pathophysiologic mechanisms underlying hemorrhage in cerebral abscesses are not yet fully elucidated. However, several hypotheses have been proposed. One theory suggests that the hemorrhage results from the rupture of fragile, newly formed vessels surrounding the abscess cavity, particularly susceptible to mechanical stress. Another possible mechanism involves the lack of thrombosis in these newly formed capillaries, rendering them prone to rupture under normal hemodynamic pressure. Moreover, oxidative stress and free radical damage to the vascular endothelium may weaken blood vessel walls, increasing the likelihood of hemorrhage. Collectively, these factors likely play a role in the development of hemorrhagic complications in cerebral abscesses, although further research is needed to clarify the exact pathways involved [7-10].

Managing cerebral abscesses generally includes a course of high-dose intravenous antibiotics for six to eight weeks. Corticosteroids and anticonvulsants, if warranted, may be used as adjunctive treatments. In cases where the lesions exceed 2.5 cm or there is a significant mass effect, more invasive treatments such as stereotactic aspiration or excision are indicated [1,2,9,10].

When treated with antibiotics and, when necessary, surgical intervention, the prognosis for a cerebral abscess is generally favorable. When left untreated, cerebral abscesses can lead to a variety of complications. These include increased intracranial pressure (ICP), which may result in brain herniation and widespread infection. Untreated abscesses can also cause neurological deficits such as seizures, cognitive impairments, motor dysfunction, and decreased levels of consciousness. Moreover, the prolonged infection can lead to irreversible brain damage, coma, and, ultimately, a mortality rate of up to 40% [1,2,4].

## Conclusions

This case highlights the need for thorough evaluation for accurate management and adherence to guidelines. Here, in our case, the patient presented with an acute course of right-sided weakness and aphasia, which may point to a vascular cause. Neuroimaging studies revealed a hyperdense lesion, which may be mistaken as a lobar hemorrhage, and upon further workup, it showed a cerebral abscess. Therefore, a careful examination, thorough history taking, and appropriate diagnostic tests are recommended to ensure early detection of the infectious culprit while starting the patient on life-saving antibiotic therapy, preventing the risk of neurologic deterioration.
